# New Trends in Immunohistochemical Methods to Estimate the Time since Death: A Review

**DOI:** 10.3390/diagnostics12092114

**Published:** 2022-08-31

**Authors:** Monica Salerno, Giuseppe Cocimano, Salvatore Roccuzzo, Ilenia Russo, Dario Piombino-Mascali, Nicholas Márquez-Grant, Christian Zammit, Massimiliano Esposito, Francesco Sessa

**Affiliations:** 1 “G.F. Ingrassia” Department of Medical, Surgical and Advanced Technologies, University of Catania, 95121 Catania, Italy; 2 Institute of Biomedical Sciences, Vilnius University, 03101 Vilnius, Lithuania; 3 Cranfield Forensic Institute, Cranfield University, Cranfield MK43 0AL, UK; 4 Department of Anatomy, Faculty of Medicine and Surgery, University of Malta, 2080 Msida, Malta

**Keywords:** immunohistochemical (IHC), immunohistochemistry, post-mortem interval, time since death, PMI, forensic pathology

## Abstract

The identification of a reliable and accurate post-mortem interval (PMI) is a major challenge in the field of forensic sciences and criminal investigation. Several laboratory techniques have recently been developed that offer a better contribution to the estimation of PMI, in addition to the traditional physical or physico-chemical (body cooling, lividity, radiocarbon dating, rigor mortis), chemical (autolysis), microbiological (putrefaction), entomological, as well as botanical parameters. Molecular biology (degradation pattern of macromolecules such as proteins, DNA, RNA), biochemical analysis of biological fluids (such as blood, cerebrospinal fluid, and vitreous humor), and immunohistochemistry are some of the most recent technological innovations. A systematic review of the literature was performed with the aim of presenting an up-to-date overview on the correlation between the immunohistochemical (IHC) expression of specific antigenic markers at different PMIs. The systematic review was performed according to PRISMA guidelines. Scopus and PubMed were used as search engines from January 1, 1998 to March 1, 2022 to evaluate the effectiveness of immunohistochemistry in estimating PMI. The following keywords were used: (immunohistochemical) OR (immunohistochemistry) AND (time since death) OR (post-mortem interval) OR (PMI). A total of 6571 articles were collected. Ultimately, 16 studies were included in this review. The results of this systematic review highlighted that IHC techniques, in association with traditional methods, add, in Bayesian terms, additional information to define a more accurate time of death and PMI. However, current IHC results are numerically limited and more data and studies are desirable in the near future.

## 1. Introduction

Determining the time since death is one of the most important aspects in the field of forensic sciences and criminal investigation [1,2,3]. The identification of a reliable and accurate post-mortem interval (PMI) is a major challenge for forensic pathologists, due to the relevant civil and criminal implications [4,5,6,7]. An erroneous PMI estimation can falsify the outcome of forensic investigations in the event of homicides, suicides, and unintentional deaths [8,9]. The term PMI refers to the time elapsed between the time of death and the discovery and examination of the body [1,2,4,10]. The estimation of the PMI can be an extremely complicated process, as it can be influenced by various endogenous and exogenous factors (environmental temperature or humidity, or health status at the time of death) [4,6]. Considering that a diverse range of variables alter the body in the post-mortem period, this research field remains challenging [7,11].

Thanatology is a science that evaluates all the macro- and microscopic post-mortem changes that occur in the human body due to lack of oxygen, anabolic processes, and cellular degradation [8,12]. PMI is usually estimated through an external and physical examination of the body, evaluating the degree of body cooling, hypostasis, rigor mortis, autolysis, and putrefaction [4,8]. As the PMI increases, it becomes more difficult to accurately estimate the time since death [8]. The post-mortem period can be classified by some authors and practitioners into early and late [2,6,10,13]: the early post-mortem period is usually estimated through physical examination of the corpse [2,7,10,14] and ends when soft tissue decomposition begins [6], commonly within the first 24 h of death [2,10]. Unfortunately, these parameters depend on the examiner’s subjective assessment [15]. The late post-mortem period ranges from one day to months or years after death; in such cases the estimation of a limited PMI range cannot be accurate [13], as the physical examination of the corpse, the evaluation of the state of decomposition, the radiometric dating of skeletal remains, and entomological studies are highly influenced by individual and environmental factors [2,7,8,10]. 

Several laboratory techniques have recently been developed that offer a better contribution to the estimation of PMI, in addition to traditional physical or physico-chemical (body cooling, lividity, rigor mortis), chemical (autolysis), microbiological (putrefaction), entomological, radiocarbon dating, and botanical parameters [4,10,13,16]. 

Molecular biology (degradation pattern of macromolecules such as proteins, DNA, RNA), biochemical analysis of biological fluids such as blood, cerebrospinal fluid, vitreous humor [5,6,8,17,18,19], and immunohistochemistry are some of the most recent technological innovations [2,4,10,20] but still have their challenges and require further validation [21]. 

Further innovative techniques for the estimation of the PMI involve the use of real-time quantitative polymerase chain reaction for the study of nucleic acids, the investigation of the human thanatotranscriptome [2,4,10], infrared microscopic imaging techniques of human skeletal remains, chemiluminescence tests, radiocarbon techniques, spectroscopical analysis, macroscopic UV fluorescence, and detection of various radionuclides [22,23,24,25,26]. One of the most studied fields to define the time since death is immunohistochemistry. Many authors have recently performed immunohistochemical (IHC) tests on human samples to evaluate the morphological changes to soft tissue that occur after death, with an attempt to identify the expression of specific markers useful for the determination of PMI [27,28,29,30]. Barrios Mello et al. [30] reported that different IHC stains could be very useful in estimating the age at death, by evaluating the preserved bone collagen fibers and endothelial cells. Intriguingly, Khalaf et al. [29] applied IHC techniques in an animal model to determine wound age and vitality.

Considering the great advances in recent years, a systematic review of the literature was performed with the aim of presenting an up-to-date overview on the correlation between the expression of specific antigenic markers at different PMIs. In this way, this overview may serve as a guide to the forensic pathologist when establishing the time since death. 

## 2. Materials and Methods

A systematic literature review was undertaken according to PRISMA guidelines [31], but the manuscript was not registered because the application is not human health but forensic sciences. Scopus and PubMed were used as search engines for publications between January 1, 1998 to May 1, 2022 to search for journal articles relating to the effectiveness of immunohistochemistry in estimating PMI. This was to summarize the main data published in the last 25 years. The following keywords were used: (immunohistochemical) OR (immunohistochemistry) AND (time since death) OR (post-mortem interval) OR (postmortem interval) OR (post mortem interval) OR (PMI).

### 2.1. Inclusion and Exclusion Criteria

The exclusion criteria used were: (1) not relevant publication (not related to the topic), (2) reviews, (3) letters or editorials, (4) articles in a language other than English, (5) animal studies. The inclusion criteria were: (1) original article, (2) articles in English, (3) human studies, (4) analysis of PMI, (5) forensic pathology report.

### 2.2. Quality Assessment and Data Extrapolation

Authors G.C. and I.R. evaluated all articles, excluding 5738 that from the title or abstract analysis were judged to be not relevant to the study. In cases of discrepancy of opinion between the inclusion or exclusion of articles, these were submitted to authors S.R. and M.E. who read the article and evaluated the criteria. In order to evaluate the degree of agreement between the studies, Kappa’s statistical test was applied [32] showing a high value (κ = 0.88). A consensus process resolved disagreements concerning eligibility.

### 2.3. Characteristics of Eligible Studies

**Figure 1:**
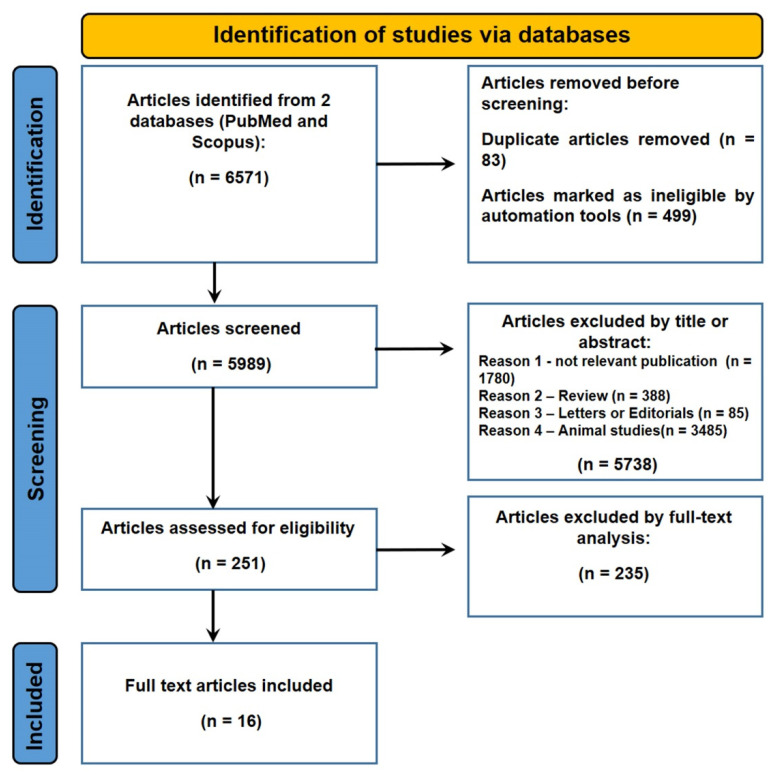
Flow diagram illustrating the studies included and excluded in this systematic literature review.

A total of 6571 articles were collected. Of these, 83 were duplicates. Of the articles that were excluded 428 concerned animals, 71 were reviews, and 5738 were not relevant (by title or abstract) to PMI; 16 studies were included in this review (Figure 1).

### 2.4. Risk of Bias

Of the 16 articles included, only 8 described the methods of preservation of the corpse. In most cases, once the subject died, the body was taken to the mortuary and stored there at a variable temperature (between 0 and 4 °C) for a maximum period of 4 days. The state of decomposition was not always described by the authors and may have influenced some of the results.

## 3. Results

The summary of all 16 articles included in this systematic review is shown in Table 1.

All cases concerned deceased persons with a PMI known through circumstantial evidence. In some studies, the method of preservation of the corpse was described, with cadavers being stored between 0 °C and 4 °C in mortuary refrigerators.

The number of cases for each article varied, with a minimum of four cases described by Ceausu et al. [41] and a maximum number of 500 described by Wehner et al. [38]. The cause of death varied, with most cases related to sudden cardiac death, traumatic brain injury, and neoplastic disease. The PMI in the studies ranged from 0 h to a maximum of 45 days [34].

**Figure 2:**
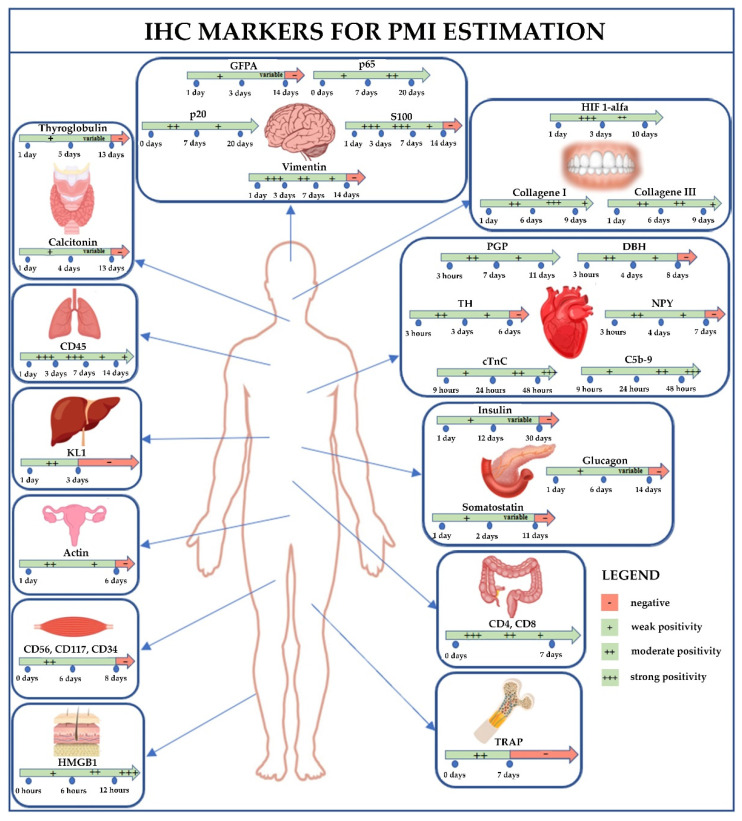
This picture summarizes the results of this review: the IHC results depend on tissue and type of immunostaining, which varies depending on the PMI. For each tissue is reported the related marker; moreover, in each box, there is an arrow: the green color indicates the interval time with positivity result, while the red color indicates the time when the marker became negative. List of abbreviations: IHC, immunohistochemical; PMI, post-mortem interval; PGP, Protein gene product; DBH, Dopamine β-hydroxylase; TH, Tyrosine hydroxylase; NPY, Neuropeptide Y; GFAP, glial fibrillary acidic protein; TRAP, Tartrate-resistant acid phosphatase; HIF 1-alfa, Hypoxia-inducible factor 1-alpha; cTnC, cardiac Troponin C; HMGB1, High-mobility group box-1; KL1, Cytokeratin 1.

The samples used to estimate the PMI came from different organs, such as brain [38,39,42], thyroid [35,36], pancreas [34,37,38], gingival tissue [14,27], skin [44], heart [33,43], large intestine [45], female genital system [46], bone marrow [40], skeletal muscle [41], as well as lung and liver [42]. For these tissues, the various authors used different markers, without any overlap or repetition (Figure 2).

Lesnikova et al. [42] published the only study in which the expression of different markers in several tissues was evaluated simultaneously. Bile duct epithelium, pulmonary lymphocytes, endothelial cells of cerebral blood vessels, glial cells, and myelin of 120 corpses were stained with anti-KL1, anti-CD45, anti-vimentin, and anti-S100 antibodies, detecting a clear positivity of all these antibodies in all tissues up to 3 days PMI. With a PMI greater than 3 days, a gradual decrease in staining rates in all tissues was observed. In brain and lung tissues, IHC staining was reliable up to 7 days. After 14 days, the only positive immunoreaction was found for the CD45 lung antigen.

Tao et al. [39] analyzed caspase-3 (p20) and nuclear factor kappa B (NF-κB) (p65) in brain nerve cells of 47 corpses (35 died following traumatic brain injury (TBI), and 8 control cases) with a PMI from 0 to 20 days. In the TBI group, caspase-3 immunostaining increased 12 h after death, concomitantly with positivity in NF-κB IHC staining. Starting from 7 days after death, the positivity of NF-κB began to increase, reaching the highest immunoreaction positivity after 20 days.

In 2006, Wehner et al. [38] published the study in which the highest number of samples was evaluated. The authors evaluated changes in IHC expression in pancreatic and brain tissue of 500 corpses through anti-somatostatin and anti-glial-acid fibrillar protein (GFAP) antibodies with a PMI between 1 and 24 days. In the pancreas, a positive somatostatin immunoreaction was detected in all cases within the first 2 days after death, while it was always negative after a PMI longer than 11 days. In brain tissue, GFAP staining was always positive within 3 days after death, becoming negative in all cases with a PMI of 14 days. 

Two further important IHC studies of pancreas tissue have been reported, using anti-glucagon and anti-insulin antibody markers. Wehner et al. [37] discovered that a positive immunoreaction to glucagon was always present in bodies with a PMI earlier than 6 days, while it was always negative with PMIs exceeding 14 days. The same authors [34] showed that insulin positive IHC staining occurred in all cases up to 12 days after death and was always negative after 30 days.

Wehner et al. [36] also studied the IHC expression of the thyroid gland in 136 cadavers through anti-calcitonin antibodies, demonstrating a positive immunoreaction of this marker up to 4 days after death in all subjects and a negative immunoreaction starting from 13 days PMI. 

Finally, the same researchers [35] used anti-thyroglobulin antibodies in thyroid tissue of 147 subjects, showing in all cases a positive immunoreaction to thyroglobulin in a PMI up to 5 days after and the absence of staining with PMIs longer than 13 days. 

Two IHC studies focused on gingival tissue. Mazzotti et al. [27] used antibody markers directed against type I and type III collagen protein in gingival tissue, detecting a strong and widely distributed signal in the group with PMIs between 1 and 3 days. In the “medium” PMI sample group (4–6 days after death), the type I collagen protein signal was higher than in the earlier group, while type III collagen positivity was less intense. In the last PMI group (7–9 days), type I collagen positivity was greatly reduced, while type III was weak, suggesting that collagen I and III positivity indicated a short and medium PMI, respectively.

Fais et al. [14] evaluated the IHC expression of the Hypoxia-Inducible Factor (HIF)1-alpha protein in 13 subjects (10 traumatic deaths and 3 control cases) at PMIs ranging between 0 and 9 days. In the traumatic deaths, the immunoreaction reached its peak positivity in the first 3 days, progressively decreasing after that. The staining was always negative in the control cases.

Other studies [33,43] have focused on the histochemical study of the heart. Elazeem et al. [43] performed IHC staining using anti-C5b-9 and anti-cardiac Troponin C (cTnC) antibodies on 70 corpses (20 from non-cardiac related fatalities and 50 deaths from firearms or stabbings resulting in heart trauma). A mild reaction to both markers was detected in all cases within 24 h after death. Between 24 and 48 h after death, there was a progressive increase in the expression of both markers, especially in the stab wound sub-group. After 48 h there was a strong positive immunoreaction, especially in the group that had cardiac lesions from violent related deaths.

Chow et al. [33] analyzed four additional antigenic myocardial markers using an anti-protein gene product (PGP), anti-dopamine β-hydroxylase (DBH), anti-tyrosine hydroxylase (TH), and anti-neuropeptide Y (NPY) in five corpses without cardiovascular disease. PGP showed a reduction in IHC expression starting on the seventh day after death, until it was reduced to about one third of the initial sample on the eleventh day. DBH began to decline on the fifth day after death, becoming progressively weaker and no longer detectable on the ninth day. The IHC expression of TH and NPY began to decline on the third and fourth days after death, respectively, and was no longer detectable on the seventh and eighth days, respectively.

El-Din et al. [44] evaluated the expression of the antigenic marker High-mobility group box-1 (HMGB1) in skin tissue up to a 24 h PMI, noting a weak positive reaction in the cytoplasm of few keratinocytes within 3 h after death and a progressive increase in immunostaining in the following hours. A strong positive reaction was present in the cytoplasm of several keratinocytes 24 h after death.

Zadka et al. [45] found that a significant reduction in the antigenic expression of CD4 and especially CD8 lymphocyte T markers occurred in the intestinal mucosa of eight cadavers with a PMI between 2 and 7 days. However, IHC expression of CD3 and CD45 did not show statistically significant changes in their study.

Olkhovsky et al. [46] showed that the IHC expression of actin in myofibroblasts, smooth muscle cells, and vessels of uterine tissue gradually decreased as the PMI increased. Immunostaining was always negative 6 days after death.

Ceausu et al. [41] evaluated the progressive changes in IHC expression of CD 56, CD 117, and CD34 in striated muscle tissue of four corpses with increasing PMI, observing that there was a positive immunostaining prior to 6 days after death, which became negative for all markers after 8 days PMI. 

Further work on 74 subjects was conducted by Boehm et al. [40], who evaluated the chronological variation of the expression of the antigenic marker Tartrate-resistant acid phosphatase (TRAP) in bone marrow. They reported that TRAP was detectable in osteoclasts with a constant intensity until the seventh day after death, after which there was a sudden and total negativity of the immunoreaction.

## 4. Discussion

The IHC analysis of tissues represents one of the main methods available in the last few decades used to evaluate the PMI, especially in cadavers with a long PMI. Conversely, traditional forensic pathology methods for estimating the time since death are mainly useful for the first few hours after death [9]. Thus, it is important to evaluate the different IHC markers that can be available to forensic pathologists to assist in the identification of the deceased and that can be used, where appropriate, in criminal investigations. Indeed, the estimation of time since death, or alternatively the age at death, could be useful information to be provided to the investigators [5,30]. The interest in immunohistochemistry for the estimation of the PMI has increased in recent years and various antigenic markers have been tested. However, despite the recent advances in IHC techniques in estimating PMI, the published scientific data is scarce and few reports with positive detection have been published. Nevertheless, the data compiled in this study show promising results. 

IHC changes and post-mortem decomposition of human tissues are dependent on the PMI and on the type of tissue analyzed, as summarized in Table 2. As previously discussed, although some authors have performed IHC studies on the same organs or tissues, it is difficult to compare the results because of the heterogeneity correlated with the different antibody markers that bind to structures, which degrade at different rates. The analysis of the 16 studies included in this systematic review showed that the different authors have evaluated and studied the microscopic tissue changes that develop with increasing PMI in 9 human tissues, using 18 different antibody markers. Most of the publications included in this systematic literature review predominantly used brain tissue, followed by pancreas, heart, thyroid, and gum. Other authors also studied additional tissues such as lung, skeletal muscle, liver, skin, bone marrow, colon, and uterus [47,48,49,50,51].

The microscopic changes of IHC expression are based on the fact that the tertiary structure of the protein antigens changes with an increase of the PMI and immunostaining becomes negative due to protein denaturation [9,34,38,52,53,54,55,56,57,58]. The development of proteolytic processes is the prerequisite for the changes in the expression of antigenic markers and greatly depends on several non-individual factors such as the environmental temperature [38] and the increase in PMI [52]. In fact, it is known that the decomposition and catabolic processes of proteins are more rapid in the hottest months of the year or in deaths from sepsis [38]. In such cases, the IHC staining can be negative at an earlier stage.

The main IHC data is derived from the work undertaken by Wehner and colleagues [34] who studied the IHC expression in the post-mortem period of various antigens including thyroglobulin and calcitonin (in thyroid tissue), insulin, glucagon, and somatostatin (in pancreatic tissue), and GFAP (in brain tissue). Specifically, through the use of anti-insulin antibodies, the authors provided important information on the microscopic changes that occur in the pancreas up to 45 days after death [34]. In pancreatic beta cells, a positive immunoreaction to insulin occurred in all cases with a PMI less than 12 days, while it was always negative with a PMI greater than 30 days. Based on this information, it can be assumed that the PMI is greater than 12 days if the immunological reaction is negative and that death occurred within 29 days in the case of positive immunoreaction.

Two years later, the same research group published two IHC studies on thyroid tissue, using anti-calcitonin [36] and anti-thyroglobulin [35] antibodies as markers; and a study on pancreatic tissue using anti-glucagon antibodies, obtaining useful results up to 14 days after death. 

In the thyroid, colloidal, and follicular cells, all cases showed a positive immunoreaction to thyroglobulin up to 5 days after death and a negative immunoreaction starting from the thirteenth day. According to Wehner et al. [35], therefore, an immunoreaction can be negative if the PMI is greater than 5 days and can be positive if the PMI is less than 13 days. Similar results were also reported using anti-glucagon antibodies in pancreatic tissue [37].

The use of anti-calcitonin antibodies also appears to give useful indications regarding the time of death [36], considering that the immunoreaction in thyroid C cells is always positive within the first 4 days after death and always negative with PMIs greater than 12 days.

In 2006, Wehner et al. [38] improved the IHC study of pancreatic tissue with anti-somatostatin antibodies, obtaining microscopic results that can be useful in the determination of PMI up to 11 days after death. In fact, a negative immunostaining to somatostatin can indicate that death occurred more than 3 days before, whilst a positive result indicates a maximum PMI of 10 days.

Brain tissue is the organ on which the greatest number of IHC studies have been performed. Wehner and Lesnikova discovered IHC antigenic patterns that could guide forensic pathologists in PMI determination up to 14 days, through the expression of GFAP [38], vimentin, and S100 [42]. In cases of negative immunostaining to GFAP, it can be interpreted that the PMI is greater than 4 days, and, on the contrary, it is possible to extend the PMI up to 13 days when the immunoreaction is positive. On the other hand, Tao [39] examined the microscopic features of brain tissue up to 20 days after death in subjects with head trauma.

Chandana et al. [59] evaluated the changes in the IHC expression of GFAP, synaptophysin, and neurofilament (NF) between 4 and 18 h after death in nerve tissue from the frontal cortex, cerebellum, caudate nucleus, and the substantia nigra of nine corpses. Unlike Wehner’s results, an increase in the expression of GFAP and NF was seen in the substantia nigra as the PMI increased. The IHC analysis of areas other than the substantia nigra did not differ from Wehner’s results and did not appear to be susceptible to significant IHC changes with increasing PMI. Such changes seem to be limited exclusively to the substantia nigra, possibly due to the dopaminergic profile of this area [59].

El-Din et al. [44] demonstrated that the skin expression of the HMGB1 protein can play an important role in estimating the PMI, because of a gradual increase in the IHC expression of the HMGB1 protein in the skin. Therefore, the detection of a strong immunoreaction to the HMGB1 antigen demonstrates that the PMI is greater than 12 h, as confirmed in both humans and rat models. 

Elias et al. [60] carried out an important analysis of the changes in the IHC expression of the p53 and bcl-2 proteins that occur in the skin tissue, at different PMIs. The authors highlighted a noticeable increase in the expression of the p53 protein in the skin that reached its maximum 6 h after death, and a slow reduction of the Bcl-2 protein due to the alteration of cellular membrane proteins resulting from the high amount of free radicals. 

Similar results to those published by Elias et al. [60] on the IHC expression of the p53 protein were reported by Mohamed et al. [61] and Khalifa et al. [62] in studies conducted on the brain tissue of rabbits and on liver, pancreatic, skin, and kidney tissues. Other work on animal models include an IHC study on dogs, where IHC staining of T cells and B cells in the cervical and bronchial lymph nodes appeared to be related with PMIs up to 23 days after death [63].

Lee et al. [55] performed an IHC study on mice, evaluating the degradation of glycogen synthase and caspase-3 in relation to PMI through the respective antibodies levels in the kidney and iliopsoas muscle [64]. The study found that it was not possible to detect glycogen synthase 48 h after death and caspase-3 after 96 h. Tao et al. [39] found that caspase-3 immunoreaction had a different pattern of expression in an animal model from that found in human brain tissue. A reduction in the IHC expression of caspase-3 in the medullary, cerebral, cardiac, and renal tissue of rats was also described by Khater [65], who carried out studies with PMI between 0 and 48 h. The results of this latest study were also in line with those reported by Lee et al. [64]. 

Elgawish [66] evaluated the IHC degradation of prostatic type III collagen to estimate the PMI in 40 male rats. A gradual reduction in the IHC expression of type III collagen was observed within 20 to 24 h after death, and was completely negative in a PMI of 60–72 h [66]. These results matched those obtained by Mazzotti et al. [27] from human gingival tissue, where the negativization of the immunostaining of type III collagen fibers was slower to develop. On the contrary, when El-Din et al. [44] performed a similar IHC study on human and mouse models, it was not clear whether the IHC changes after death observed in animals were equivalent to those seen in humans. Despite the fact that animal models are crucial for initial scientific experimentation, extrapolation of data to human models is not always fully applicable due to differences between the two species in terms of anatomical, physiological, biochemical, and genetic diversity [59,67,68,69,70,71].

Regarding the correlation between PMI and other variables, some authors highlighted that the IHC changes of tissues can also be influenced by individual factors and by the cause of death. Indeed, Tao [39], Elazeem [43], and Fais [14] have shown that the IHC changes that develop after death are different in trauma-induced deaths, when compared to control cases. Cho et al. described specific markers to estimate the PMI in cases of drowning in murine models [72] with a PMI from 1 to 7 days, demonstrating that the IHC expression of the mRNA receptor of Advanced Glycation End Products (RAGE) gradually decreases as the PMI increases.

Studies correlating IHC expression changes and PMI are often performed under constant temperature and well-established conditions. A limitation of the studies reported in the literature could therefore be the lack of variable environmental conditions on the degradation rate of different tissues. Furthermore, most of the studies carried out never exceeded a PMI of more than 45 days. In light of these considerations, future research should aim to fill this gap by performing studies exploring different environmental/burial conditions. Considering the importance of PMI estimation, it would be ideal if future studies combined IHC techniques with promising postmortem proteomics tools in order to discover sensitive and specific biomarkers that can be used for forensic applications. Recent data provide strong evidence in favor of the applicability of a protein degradation-based PMI estimation method in routine forensic practice [8]. In particular, different proteins, such as eEF1A2 and GAPDH, seem to be valuable markers [73]. Nevertheless, a significant limiting factor is the ability to stop the tissue degradation process at the time of sampling. One way to circumvent this limitation is by snap-freezing and storing the samples in liquid nitrogen, but this procedure is impractical for routine application [74]. Further studies are thus needed to evaluate the applicability of these new techniques for day-to-day forensic cases.

The main strengths of this review include the high value for the Kappa’s statistical test based on the agreement between the studies, the search methodology, and a flowchart describing in detail the study selection process. On the other hand, several limitations may be attributed to this review, including: the selected keywords may have influenced the search strategy; despite the application of several countermeasures, this review may suffer from the influence of the authors’ personal viewpoints; the presence of gaps in literature searching practices that may be related to the use of the selected databases; and the difficulty in summarizing IHC data biased by the pathologist’s interpretation.

## 5. Conclusions

In the last few years, many authors have performed IHC tests on human samples to evaluate the morphological changes that occur after death, trying to identify the expression of specific markers useful for the determination of the PMI. The results of this systematic literature review highlighted that IHC techniques, in association with traditional methods, add, in Bayesian terms, further information useful to define a more accurate time of death and PMI. Immunohistochemistry can estimate the time since death and the PMI more accurately when compared with traditional methods, which could be dependent on examiner bias, a PMI that is too large, or contradictory results. However, it is important to perform further studies on IHC analysis, particularly with well-defined experimental conditions. In fact, the IHC results are different according to the PMI and depend on the antibody used. It appears that some antigens become negative after a few hours, and others after several days. To estimate the PMI, it is useful to evaluate the degree of antigenic expression of the tissues to the different markers. Current IHC results are numerically limited and more data and studies are clearly needed. In the future, researchers should further test antibodies capable of consolidating data already acquired as well as providing additional information, especially in cadavers that have a late PMI and, moreover, paying careful attention to the various individual and environmental variables, and to the manner of death.

## Figures and Tables

**Table 1:** Summary of the results of the systematic review.

Reference	n. of Cases (Cause of Death)	Sample	Marker	PMI	Findings
Chow et al., 1998 [33]	5 (cerebral infarction, nasopharyngeal carcinoma, cerebral infarction, carcinoma of esophagus, malignant lymphoma)	Heart	Protein gene product (PGP), Dopamine β-hydroxylase (DBH), Tyrosine hydroxylase (TH), Neuropeptide Y (NPY)	From 3 days to 6 days	Reduction of the IHC expression of TH
				From 4 days to 7 days	Reduction of the IHC expression of NPY
				From 4 days to 8 days	Reduction of the IHC expression of DBH
				From 7 days to 11 days	Reduction of the IHC expression of PGP
				From 6 days to 11 days	Absence of detection of TH
				From 7 days to 11 days	Absence of detection of NPY
				From 8 days to 11 days	Absence of detection of DBH
				11 days	PGP is reduced to about one third of the initial value
Wehner et al., 1999 [34]	128	Pancreas	Insulin	From 1 day to 12 days	Positive immunoreaction in all cases
				From 13 days to 29 days	Variable situation concerning the immunoreaction. Some cells are positive others negative
				From 30 days to 46 days	Always negative immunostaining
Wehner et al., 2000 [35]	147	Thyroid	Thyroglobulin	From 1 day to 5 days	The thyroid gland colloid and follicular cells present a positive immunoreaction in all cases
				From 6 days to 12 days	Variable situation concerning the immunoreaction. Some cells are positive, others negative
				From 13 days to 22 days	Always negative immunostaining
Wehner et al., 2001 [36]	136	Thyroid	Calcitonin	From 1 day to 4 days	The thyroid gland c-cells are positive in all cases
				From 5 days to 12 days	Variable situation concerning the immunoreaction. Some cells are positive, others negative
				From 13 days to 22 days	Always negative immunostaining
Wehner et al., 2001 [37]	214	Pancreas	Glucagon	From 1 day to 6 days	Positive immunoreaction in all cases
				From 7 days to 13 days	Variable situation concerning the immunoreaction. Some cells are positive others negative
				From 14 days to 22 days	Always negative immunostaining
Wehner et al., 2006 [38]	500	Pancreas and brain	Somatostatin and anti-glial fibrillary acidic protein (GFAP)	From 1 day to 2 days	Positive somatostatin immunoreaction in all subjects
				From 1 day to 3 days	Positive GFAP immunoreaction in all subjects
				From 3 days to 10 days	Variable positive somatostatin immunoreaction
				From 4 days to 13 days	Variable positive GFAP immunoreaction
				From 11 days to 24 days	Negative immunoreaction to somatostatin in all subjects
				From 14 days to 24 days	Negative immunoreaction to GFAP in all subjects
Tao et al., 2006 [39]	47 (39 traumatic brain injury -TBI, 8 non-traumatic)	Brain	Caspase-3 (p20) and NF-κB (p65)	0 h	A few positive cells in NF-kB (p 65) IHC staining after TBI. A few positive neurons in caspase-3 (P20) IHC after TBI
				12 h	A few positive cells in NF-kB (p 65) IHC staining after TBI. Much more positive neurons in caspase-3 (P20) IHC after TBI
				24 h	Caspase-3 (p20) positivity became darker after TBI
				48 h	Most neurons are positive for caspase-3 (p20). Reduction in the number of caspase-3 (P20) immunohistochemistry positive cells after TBI
				72 h	Increased immunoreaction to caspase-3 of neurons and especially of glial cells after TBI
				168 h	Strong positive NF-kB (p 65) IHC staining after TBI
				264–480 h	Increases the cellular IHC expression of caspase-3 after TBI. The endothelium of all groups showed a positivity to caspase-3
				480 h	Almost all cells are NF-kB (p 65) positive after TBI
Boehm et al., 2012 [40]	96 (cardiovascular, metabolic or respiratory failure, septic shock, trauma, intoxication, cancer and 22 control cases)	Bone Marrow	Tartrate-resistant acid phosphatase (TRAP)	<7 days	Positive immunostaining in osteoclasts
				>7 days	Usually negative immunostaining
Ceausu et al., 2016 [41]	4	Skeletal muscle	CD56, CD117 and CD34	From 1 day to 6 days	Staining positive for CD56, CD117 and CD34
				8 or more days	Absent immunostaining
Lesnikova et al., 2018 [42]	120	Liver, lung, and brain	KL1 (bile duct epithelium), S100 (glial cells and myelin), vimentin (cerebral endothelial cells), and CD45 (pulmonary lymphocytes)	From 1 day to 3 days	Strong positive staining in several tissues with all antibodies
				From 3 day to 7 days	Slight decrease in staining rates of vimentin in brain tissue and absence of PCK immunoreaction in liver tissue
				From 7 day to 14 days	Significant decreased staining rates of all antibodies in several tissues
				14 days or more	IHC positivity for CD45 lung antigen only
Fais et al., 2018 [14]	13 (10 traumatic and 3 control cases)	Gingival tissue	HIF 1-alfa	From 1 day to 3 days	Immunostaining was peaked in traumatic group and absent in control group
				From 4 days to 5 days	Immunostaining gradually declined in traumatic group
				From 8 days to 9 days	Immunostaining gradually declined in traumatic group
Mazzotti et al., 2019 [27]	10	Gingival tissues	Type I and type III collagen	From 1 day to 3 days	Strong positive immunostaining of both proteins
				From 4 days to 6 days	Slight increase in IHC expression of type I collagen. Immunoreaction of type III collagen is stable
				From 7 days to 9 days	Marked reduction of cellular IHC expression of type I collagen (no signal detected in the extracellular matrix) and slight reduction of type III collagen
Elazeem et al., 2021 [43]	70 autopsies (20 non cardiac, 24 stab firearm cardiac injury, 26 firearm cardiac injury)	Heart	C5b-9 and cTnC	From 9 h to 24 h	In all groups, mild positive immunoreaction of both markers, especially cTnC
				From 24 h to 48 h	In all groups, moderate positive immunoreaction of both markers, especially of C5b-9 in the stab wound group
				More than 48 h	In all groups, severe diffuse positive immunoreaction of both markers, especially of C5b-9 in the stab wound group and in firearm injury group and cTnC in stab wound
El-Din et al., 2021 [44]	40 cadavers	Skin	HMGB1	From 0 h to 3 h	Weak positive immunoreaction in few keratinocytes
				From 3 h to 6 h	Mild positive immunoreaction in numerous keratinocytes
				From 7 h to 12 h	Moderate positive immunoreaction in numerous keratinocytes
				From 12 h to 24 h	Strong positive reaction in numerous keratinocytes
Zadka et al., 2021 [45]	24 (8 sudden death, traffic accident and 16 control cases)	Colonic mucosa	CD45, CD4, CD8, CD3	From 0 day to 7 days	Progressive and significant reduction of IHC expression of CD4 and especially of CD8
Olkhovsky, et al. 2021 [46]	48 (42 miscellaneous, 6 control cases)	Uterus	Actin	From 24 h to 48 h	Slight decrease in IHC expression
				From 49 h to 72 h	Decreased IHC expression. In few fields of view, absence of expression
				From 73 h to 96 h	Sharply decreased IHC expression
				From 97 h to 120 h	Only a few muscle cells are positive for immunostaining
				From 121 h to 144 h	Single smooth muscle cells were positive for immunoreaction. In a significant number of smooth muscle cells, the immunostaining was not detected
				More than 144 h	The expression of smooth muscle actin was not determined

**Table 2:** IHC markers in relation to the presumed PMI. Each antigenic marker undergoes changes in IHC expression associated with the PMI. After estimation of the time since death through traditional methods, it is necessary to use the most suitable IHC markers to estimate the PMI as accurately as possible. An IHC analysis can be performed for any suspected PMI, especially in the first 2 weeks after death. Abbreviations: PGP, Protein gene product; DBH, Dopamine β-hydroxylase; TH, Tyrosine hydroxylase; NPY, Neuropeptide Y; GFAP, glial fibrillary acidic protein; NF-κB, nuclear factor kappa B; TRAP, Tartrate-resistant acid phosphatase; HIF 1-alfa, Hypoxia-inducible factor 1-alpha; cTnC, cardiac Troponin C; HMGB1, High-mobility group box-1.

Presumed PMI	Tissue	IHC Marker (Staining + or −)
1–2 days	Pancreas	Somatostatin (+)
1–3 days	Brain	GFAP (+)
	Bile duct epithelium	KL1 (+)
	Glial cells and myelin	S100 (+)
	Cerebral endothelial cells	Vimentin (+)
	Pulmonary lymphocytes	CD45 (+)
1–4 days	Thyroid	Calcitonin (+)
1–5 days	Thyroid	Thyroglobulin (+)
1–6 days	Pancreas	Glucagon (+)
	Skeletal muscle	CD56, CD117 and CD34 (+)
1–7 days	Bone marrow	TRAP (+)
1–12 days	Pancreas	Insulin (+)
>6 days	Heart	TH (−)
	Uterus	Actin (−)
>7 days	Heart	NPY (−)
	Bone marrow	TRAP (−)
>8 days	Heart	DBH (−)
	Skeletal muscle	CD56, CD117 and CD34 (−)
>11 days	Pancreas	Somatostatin (−)
>13 days	Thyroid	Calcitonin (−)
		Thyroglobulin (−)
>14 days	Pancreas	Glucagon (−)
	Brain	GFAP (−)
	Bile duct epithelium	KL1 (−)
	Glial cells and myelin	S100 (−)
	Cerebral endothelial cells	Vimentin (−)
>30 days	Pancreas	Insulin (−)

## Data Availability

Data sharing not applicable, no new data were created or analyzed in this study.
